# The Social Competence Promotion Program among Young Adolescents (SCPP-YA) in Chile (“Mi Mejor Plan”) for substance use prevention among early adolescents: study protocol for a randomized controlled trial

**DOI:** 10.1186/s13063-022-06472-w

**Published:** 2022-06-30

**Authors:** Jorge Gaete, Constanza Inzunza, Saray Ramírez, Daniela Valenzuela, Cristian Rojas, Ricardo Araya

**Affiliations:** 1 Research Center for Students Mental Health (ISME), Faculty of Education, Universidad de los Andes, Santiago, Chile; 2 Millennium Nucleus to Improve the Mental Health of Adolescents and Youths, Millennium Science Initiative, Santiago, Chile; 3 Faculty of Psychology, Universidad de Talca, Talca, Chile; 4 Department of Health Service & Population Research, King’s College London, London, UK; 5 David Goldberg Centre, Denmark Hill, London, UK

**Keywords:** Substance use, Adolescents, Prevention, Schools, Randomized control trial

## Abstract

**Background:**

Substance use is highly prevalent among children and adolescents in Chile, and it is known how it impacts their health and social adjustment. The call for effective prevention of substance use among children adolescents has resulted in numerous school-based programs, and particularly, the Social Competence Promotion Program among Young Adolescents (SCPP-YA) has been proved to be successful for promoting social and problem-solving skills in addition to preventing substance abuse in the US population. The purpose of this study is to test the effectiveness of the Social Competence Promotion Program among Young Adolescents (SCPP-YA) in Chile (“Mi Mejor Plan”).

**Methods:**

This is a cluster randomized controlled trial, parallel-group type, where “Mi Mejor Plan” is compared to standard school preventive curricula in control schools. A total of 10 schools and 600 adolescents are expected to be recruited and randomized with 1:1 allocation. During formative work, the SCPP-YA program was culturally adapted to Chile. The effectiveness of this program will be assessed using the European Drug Addiction Prevention Trial Questionnaire (EU-Dap), measuring substance use prevalence and risk and protective factors in baseline, post-intervention, and 4 months after the end of the intervention.

**Discussion:**

The proposed study will be the first to test the effectiveness of the Social Competence Promotion Program among Young Adolescents (SCPP-YA) in Chile in a cluster randomized control trial and also the first study evaluating this program in Spanish-speaking Latin America. SCPP-YA has been implemented successfully in the USA. Thus, if the effects of the program are positive, wide implementation in Chile and Latin American countries is possible soon.

**Trial registration:**

Clinical Trials NCT04236947. Registered on January 22, 2020.

## Administrative information

Note: the numbers in curly brackets in this protocol refer to SPIRIT checklist item numbers. The order of the items has been modified to group similar items (see http://www.equator-network.org/reporting-guidelines/spirit-2013-statement-defining-standard-protocol-items-for-clinical-trials/).

**Table:** 

Title {1}	The Social Competence Promotion Program among Young Adolescents (SCPP-YA) in Chile (“Mi Mejor Plan”) for substance use prevention among early adolescents: study protocol for a randomized controlled trial.
Trial registration {2a and 2b}.	Trial identifier NCT04236947 in Clinical Trials [ClinicalTrials.gov] under the registry name: “Randomized Control Trial of the Effectiveness of the Social Competence Promotion Program for Young Adolescents Aimed at Preventing Substance Use Among Students in Chile”; [registered on 17-01-2020]
Protocol version {3}	Version 1 of 04-03-2021.
Funding {4}	This research is funded by the National Research and Development Agency [ANID]. Unique ID: FONIS SA19I0115. And by ANID – Millennium Science Initiative Program – NCS2021_081.
Author details (5a}	J. Gaete: Research Center for Students Mental Health (ISME), Faculty of Education, Universidad de los Andes. Chile. Millennium Nucleus to Improve the Mental Health of Adolescents and Youths, Millennium Science Initiative, Chile. C. Inzunza: Research Center for Students Mental Health (ISME), Faculty of Education, Universidad de los Andes. Chile. S.Ramirez: Research Center for Students Mental Health (ISME), Faculty of Education, Universidad de los Andes. Chile. Millennium Nucleus to Improve the Mental Health of Adolescents and Youths, Millennium Science Initiative, Chile. D. Valenzuela: Research Center for Students Mental Health (ISME), Faculty of Education, Universidad de los Andes. Chile. C. Rojas: Faculty of Psychology, Universidad de Talca, Talca, Chile. R. Araya: Department of Health Service & Population Research, King’s College London, London, United Kingdom. David Goldberg Centre, Denmark Hill, London, United Kingdom. Millennium Nucleus to Improve the Mental Health of Adolescents and Youths, Millennium Science Initiative, Chile.
Name and contact information for the trial sponsor {5b}	Investigator initiated clinical trial; Jorge Gaete (Principal Investigator). Contact Information: E-mail: jgaete@uandes.cl; Phone +56226182277; Address: Avenida Monseñor Álvaro del Portillo 12.455. Las Condes. Santiago, Chile
Role of sponsor {5c}	Funders played no role in the design of the study, collection, management, analysis, and interpretation of the report. They will not have ultimate authority over any of these activities.

## Introduction

### Background and rationale {6a}

Adolescence is a particularly challenging stage during which young people must deal with their biological development and social demands from their peers, family, and environment. These demands often produce interpersonal stress and a variety of issues that make adolescents susceptible to emotional and behavioral problems [1]. Substance abuse is especially worrying due to its implications for personal health and social adjustment. For instance, marijuana use can increase the likelihood of respiratory issues [2]. Substance use has also been found to affect cognitive functioning, with users of tobacco, alcohol, and marijuana scoring more poorly in executive function and academic performance tests [3–5]

In Chile, the latest National Study of Substances in School Population (2017) [6] has highlighted the severity of this situation. This nationwide study [6] revealed that the average age of onset of tobacco, alcohol, and marijuana use is between 13 and 14 years. These figures are concerning because scientific evidence indicates that the earlier the onset, the more devastating the impact on physical, neurological, and psychological development and the higher the risk of suffering addictive disorders in adulthood [5, 7]. Furthermore, within the month before the survey [6], 20% of the students (from 8th grade to 12th grade) reported using tobacco, about 33% reported using alcohol, and 20% reported using marijuana. Although tobacco use has been declining, alcohol use in the school population has remained relatively stable. In contrast, the annual prevalence of marijuana use has increased by 20% compared to the first survey in 2001.

In Chile, multiple substance use prevention initiatives aimed at adolescents have been evaluated and implemented. In the 1970s, the Ministries of Education and Health developed a program that was unsuccessful [8]. Over the past 30 years, the Government has promoted several initiatives, first through the National Council for Narcotics Control (CONACE) and, since 2011, through the National Service for the Prevention and Rehabilitation of Substance and Alcohol Use (SENDA). CONACE developed a “Prevention Program” consisting of various universal interventions for substance use prevention. In 2009, CONACE implemented the “Continuo Preventivo” program in schools [9]. Subsequently, in 2011, SENDA launched the “Chile Previene en la Escuela” program. Today, the “Continuo Preventivo” continues to be used, including programs between preschool and 12th grade [10]. .Another initiative is the “Familias Fuertes: Amor y Límites” program, developed by the Pan American Health Organization (PAHO). This program was implemented and evaluated in Chile through a quasi-experimental design, but six months later, adolescent involvement in risky behaviors was not reduced [11]. Despite the efforts made by the Government of Chile and several other institutions over the past few years, the results are somehow frustrating. For example, the National Plan for Substances and Alcohol 2011–2014 [12] sought to reduce the annual prevalence of marijuana and alcohol use in the school population by 15%. However, the annual prevalence of marijuana use increased dramatically from 19.5% in 2011 to 34.2% in 2015, while the annual prevalence of alcohol increased from 59.3% in 2011 to 63% in 2015 [6]. As previously noted, initiatives to prevent substance use in adolescents have been implemented but have yet to be evaluated through randomized controlled trials. For this reason, in Chile, it is necessary to evaluate programs to prevent substance use in adolescents to address this risky behavior and reduce its negative consequences.

Internationally, there are several substance use prevention programs. The Life Skills Training Program (LST) is a multi-component preventive intervention primarily intended for students to develop substance resistance skills by learning self-management strategies, establishing healthy relationships, and engaging in responsible decision-making [13]. Several RCTs have shown effectiveness in reducing tobacco, alcohol, and marijuana use among students receiving LST compared to the control group [13]. Another such program is “Project Northland” [14], a school intervention focusing on substance use prevention. It was created to be applied in rural settings characterized by problematic alcohol consumption. Results have been positive in reducing alcohol consumption and discouraging new alcohol users [14]. However, when this intervention was applied in other settings (urban, without problematic consumption), no changes in alcohol consumption were observed in adolescents [15]. The Lion’s Quest, Skills for Adolescence Program aims to develop socio-emotional competencies in students from preschool to 12th grade and includes a Substance Prevention Module [16]. This program has been evaluated in several countries, successfully reducing adolescents’ substance use while also helping them to establish healthy interpersonal relationships, increasing their motivation for learning, and reducing dropout, among other positive outcomes [16, 17]. For its part, the Seattle Social Development Project Program focuses on protective and risk factors as a way of helping students to develop interpersonal skills and better problem-solving strategies [18]. It has been evaluated through quasi-experimental studies, which have revealed less involvement in criminal acts, lower strong alcohol consumption, increased school membership, and better academic performance [19]. In a similar vein, the Raising Healthy Children Program is a multi-component preventive intervention designed to reduce risk factors and promote protective factors in student development [20]. The intervention has improved participants’ academic performance, reduced the frequency of alcohol and marijuana use (but without reducing the number of new users), and increased their school membership [18, 20, 21]. Finally, the Unplugged program [22], evaluated in seven countries (Austria, Belgium, Germany, Greece, Italy, Spain, and Sweden), is among the successful European interventions. The curriculum—which comprises 12 Unplugged sessions—is based on the theory of cognitive social influence and promotes intrapersonal skills (critical thinking versus normative beliefs) and interpersonal skills (assertiveness and skills of how to resist peer pressure), while also providing information on the different substances, in such a way as to promote good decision-making. The evaluation study showed a significant reduction in alcohol and marijuana use that persisted up to 15 months after the end of the intervention [22, 23]. This type of intervention has also allowed students to develop a better bond and a greater sense of belonging to their school [24]. A greater sense of school membership has been reported to increase involvement in healthy behaviors and reduce the likelihood of developing risky behaviors [25]. For example, a strong sense of school membership has been associated with increased motivation and academic performance [26] and greater development of health-promoting behaviors [27]. In contrast, a low level of school membership has been associated with increased negative emotions [28], suicidality [29], dropout [30], behavioral problems at school [31], and the use of substances of abuse [32].

The drawbacks or limitations of many of the programs mentioned above are: payment of a usage license and the need for a costly certified training program, a lack of validated Spanish versions for implementation in Chile or other Latin American countries, and sometimes the unwillingness of the original authors to make cultural adaptations to programs that have been tested with randomized controlled studies. The payment of the license, which only covers the use of the program without considering the costs of the materials, training, and work of teachers or facilitators, limits the viability of local implementations. Furthermore, the need to generate Spanish versions adapted to the national context represents an additional cost in terms of money and time that the authors of the programs are not always willing to cover.

Roger Weissberg’s New Haven Adolescent Socio-Emotional Development Program [33, 34], also known as the Social Competence Promotion Program for Young Adolescents, has been successfully implemented in numerous schools in the USA, especially in areas with greater economic vulnerability. This program has been implemented in several versions, each with a different number of sessions. The adaptation made by the research team behind this project comprises 16 sessions delivered to students by trained teachers throughout the academic year and is specially designed for students of an educational level equivalent to 6th grade. The program has two modules: The problem-solving skills module and the Substance Use prevention module. The first module has 11 sessions focused on practicing several problem-solving skills using interactive methodologies. The second module has five sessions centered on learning how to prevent substance use and how the skills learned in the problem-solving module can be applied to prevent drug use. In addition, it has three reinforcement sessions for the following academic year, also delivered by a trained teacher. A randomized controlled trial showed that the program had several beneficial effects for young teens: it allowed them to acquire problem-solving skills, improved their social relationships with peers, reduced their behavioral problems and alcohol consumption, and decreased the development of criminal behaviors [35]. In terms of problem-solving capacity, students who participated in this program improved in terms of the quantity and quality of their problem-solving choices compared to the control group. Positive engagement with peers also increased due to the program [36]. This program has been selected by several agencies such as the Oregon Addiction and Mental Health Services, the Washington Division of Behavioural Health and Recovery [37], and the Centre for Disease Control and Prevention of the Department of Health and Human Services [37] as a model program for promoting social and problem-solving skills in addition to preventing substance abuse. The authors of this project have contacted Roger Weissberg, who granted his authorization to adapt this program to the Chilean context and evaluate its effectiveness in Chile. Among its advantages compared to other programs, we can mention the following: (1) it does not require a paid usage license, which makes it possible to project the beneficial effects of the program at a low cost when scaling it to a larger population; and (2) this program has been widely used in populations of high socioeconomic vulnerability, including African-American, Latino, and Caucasian populations, which supports its applicability to similar populations in Chile. In this study, we will adapt the Social Competence Promotion Program among Young Adolescents (SCPP-YA) for schools in Chile (henceforth in Chile “Mi Mejor Plan”), evaluating its effectiveness in this new context. Besides its practical significance, the project will contribute to the scientific discussion on the replicability of substance use school-based prevention programs in new contexts.

### Objectives {7}

The main objective of this study is to develop a culturally appropriate version of an evidence-based substance use prevention program called Social Competence Promotion Program among Young Adolescents (SCPP-YA), adapted to the Chilean culture (Mi Mejor Plan), and to test its effectiveness among early adolescents in primary schools in Santiago, Chile.

There are two stages in this study, formative work and the study of the effectiveness of the program. The specific objectives regarding formative work are the following: (1) to translate and culturally adapt the SCPP-YA program and its Substance Use Prevention Module to the context of Chile. This work was conducted during 2019–2020; (2) to create a training course for the future facilitators. The final stage is conducting a Cluster Randomized Controlled Trial to test the effectiveness of the program. The specific objective of this stage is (1) to compare the level of self-reported substance use of 6th graders participating in the “Mi Mejor Plan” program and control schools immediately after the end of the intervention and 4 months later, controlling by baseline assessment; (2) to assess the acceptability and feasibility of the intervention in the context of schools in Chile. This study will be carried out in 2022 and 2023, presenting its protocol here.

We hypothesize that at the end of the intervention, adolescents who received the “Mi Mejor Plan” program, when compared to standard school drug prevention curricula, will have a lower 1-month of cigarette, alcohol, and marijuana use.

### Trial design {8}

**Fig. 1:**
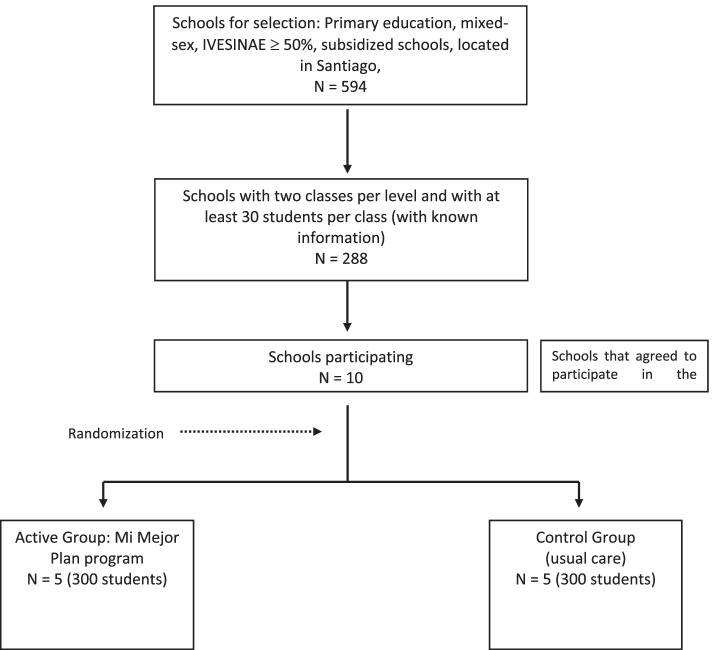
Flowchart

This is a cluster randomized controlled trial, parallel-group type, where the “Mi Mejor Plan” program is compared to standard school prevention curricula in control schools. The standard school of drug prevention curriculum is delivered in the orientation class, henceforth “Treatment-As-Usual” (TAU) [38], and it will be used as a comparator to determine the superiority of “Mi Mejor Plan” over TAU in Chile. The patient allocation is randomized with a ratio of 1:1 (See Fig. 1).

## Methods: Participants, interventions, and outcomes

### Study setting {9}

This trial will be performed in primary schools located in Santiago, Chile. There are 2918 schools in Greater Santiago (Region Metropolitana). After applying the inclusion criteria, the total number of eligible schools was 288. All eligible schools will be contacted, and the first 10 schools interested in participating in the study will be included in the project. These schools will be representative of the most vulnerable schools in Chile.

### Eligibility criteria {10}

#### Inclusion criteria for schools

- Schools located in Santiago (Chile)
- Schools of Primary Education (1st grade to 8th grade)
- Mixed-sex schools.
- Schools with at least two classes in the 6th grade.
- Schools with at least 30 students per class
- Schools with vulnerability (≥50%), measured with the School Vulnerability Index – National System of Equality Allocation (IVE-SINAE). This index is the proportion of students in a given school with high vulnerability. This index considers the following socioeconomic variables to group the schools: mother’s educational level, father’s educational level, and total monthly household income, among others [39].

#### Exclusion criteria for schools

Schools implementing other substance use prevention programs similar to the contents and methodology of “Mi Mejor Plan” delivered to the same grade.

#### Inclusion criteria for participants

- Attending 6th.

### Who will take informed consent? {26a}

Schools will be screened for eligibility to participate in this study based on the aforementioned criteria. After the schools have been assessed as eligible by research assistants, the principal of the selected schools will receive study information. After the school authorities accept participating in the study and sign a form, parents of students will be informed about the study and that the “Mi Mejor Plan” program will be part of the school curriculum sending information letters to their homes. Along with this information, the parent will receive the Informed Consent Form, and they need to sign it if they agree that their children participate in the study. Finally, the students will be informed about the study by research assistants and asked to sign an assent confirming their participation.

### Additional consent provisions for collection and use of participant data and biological specimens {26b}

Not applicable. No biological samples collected.

## Interventions

### Explanation for the choice of comparators {6b}

The control group receives standard school drug prevention curricula (“Treatment-As-Usual,” TAS) [38], which is not a manualized intervention. The standard curriculum includes a class called “Orientation,” where students usually participate every week in a class where they receive teaching on health promotion and substance use preventive messages. Most of the material used in these classes comes from state agencies, such as the Ministry of Education and the National Service for the Prevention and Rehabilitation of Drug and Alcohol Abuse (SENDA). Moreover, the curriculum of this class usually does not follow a manualized intervention such as “Mi Mejor Plan”; therefore, we chose the usual orientation class as a treatment-as-usual comparator to determine the superiority of “Mi Mejor Plan” over the common practice in Chile.

### Intervention description {11a}

The intervention group will receive the “Mi Mejor Plan” program. This program will be implemented during the academic year during school hours, mainly in the “Orientation” class. The program’s curriculum consists of sixteen weekly lessons delivered by a trained facilitator (member of the research team) accompanied by a school-designated teacher (who also will be trained). It is divided into two modules: (1) a social problem-solving module, consisting of 11 lessons on a method for solving social problems, and (2) a substance use prevention module, consisting of five lessons on how to apply the problem-solving method to help adolescents to prevent using substances. The research team had design-specific material for facilitators and teachers, as well as for students, to help them to complete the program. The research team will provide all the material, training, and coaching during the study. The research team will deliver the 2-day training to all facilitators, teachers, and participating school staff at the beginning of the academic year. After all the 16 lessons are completed, three booster sessions will be delivered 6–12 months later.

### Criteria for discontinuing or modifying allocated interventions {11b}

As the program is part of the school curriculum, students in the intervention group will participate in all sessions of the program. Even though they cannot leave the classroom if they do not want to participate in the sessions, they can leave the study for any reason if they wish to do so without any consequences. This means that their information and collected data will not be analyzed. On the other hand, the control group will keep its condition during the whole trial.

### Strategies to improve adherence to interventions {11c}

Adherence to the “Mi Mejor Plan” will be monitored by research assistants from the research team. After each session, teachers will fill out a survey informing students’ attendance and information about the quality of the delivery. Research assistants will be in close contact with school authorities and teachers, monitoring the progression of the program during study visits.

### Relevant concomitant care permitted or prohibited during the trial {11d}

The exclusion criterion states that schools implementing other substance use prevention programs similar to the contents and methodology of “Mi Mejor Plan,” delivered to the same grade, cannot be included in the study. This decision is based on the potential contamination of the results if another similar program is implemented in the control group. This study aims to test the effectiveness of “Mi Mejor Plan” over TAU in Chile.

### Provisions for post-trial care {30}

There is no potential harm or damage in this trial. The intervention group will receive an evidence-based intervention, and the control group will receive the usual prevention curriculum.

### Outcomes {12}

#### Primary outcome

Cigarette use in the last month, measured with the European Drug Addiction Prevention Trial Questionnaire (EU-Dap) validated in Chile [40].

#### Secondary outcomes

1. Alcohol use in the last month, measured with the European Drug Addiction Prevention Trial Questionnaire (EU-Dap) validated in Chile [40].
2. Marijuana use in the last month, measured with the European Drug Addiction Prevention Trial Questionnaire (EU-Dap) validated in Chile [40].
3. Social problem-solving, measured with The Social Problem-Solving Inventory-Revised (SPSI-RS) abbreviated 25-item version [41].
4. Emotion regulation, measured with the Emotion Regulation Questionnaire for Children and Adolescents (ERQ-CA) [42, 43].
5. Socio-emotional skills measured with the Strengths and Difficulties Questionnaire (SDQ) answered by students [44].
6. Sense of school membership, measured with the Psychological Sense of School Membership Scale (PSSM), answered by students [45].

### Participant timeline {13}

Table 1 shows the participant’s timeline.

**Table 1:**
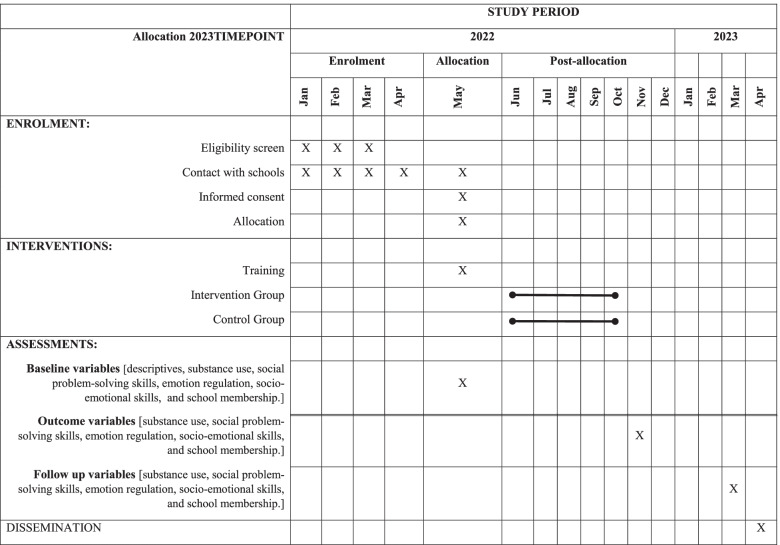
Standard Protocol Items: Recommendations for Interventional Trials (SPIRIT) diagram

### Sample size {14}

For the sample size calculation, an absolute difference between the control group and the intervention group of 15% in the monthly cigarette consumption was adopted. Since students in the same educational institution show correlated behaviors, it is important to consider the nested data within the school for sample calculation. For substance use behaviors, the intraclass correlation coefficient (ICC) can be set at 0.01. Thus, with a difference between groups of 15%, statistical power of 80%, a significance level of 0.05, an ICC of 0.01, and a school size of about 50 students per school, four educational establishments, and 200 students per data collection branch are required. Considering a loss of 20% of the schools in the follow-up, we have included one more school in each arm, ending up of five schools and 300 students per arm.

### Recruitment {15}

The strategies for achieving adequate school enrolment to reach the target sample size will include contacting and presenting the study municipality authorities who are expected to help establish contact with school authorities. Additionally, research assistants will contact directly and inform school authorities about the study’s purpose, requirements, and duration. Our research team already has good collaborative networks with municipalities and schools in Chile.

## Assignment of interventions: allocation

Randomization will be performed once all schools are recruited. Using computer-generated randomization, schools will be randomly assigned to either group with a 1:1 allocation.

### Sequence generation {16a}

Randomization will be performed once all schools are recruited. Using computer-generated randomization, schools will be randomly assigned to either group with a 1:1 allocation.

### Concealment mechanism {16b}

After the randomization and allocation, schools will be informed by a research assistant of the group of belonging by email and confirmed by telephone. Each school will only receive their information of allocation. Additionally, this information will not be disclosed to the assessment research team (outcome evaluators) to keep the blind to the schools’ allocation.

### Implementation {16c}

An independent statistician will perform the randomization to assign to the study arms, and this statistician will give this information to a research assistant. Later, the research assistant will inform schools by email and confirm by telephone.

## Assignment of interventions: blinding

### Who will be blinded {17a}

This is a double-blinded trial, blinded to the outcome evaluators and the data analyst. Outcome evaluators will not be informed about the group of belonging and will be instructed not to ask the students or the school authorities about the school condition. A data analyst will work with the final dataset where the group condition will be masked.

### Procedure for unblinding if needed {17b}

The design is open label with only outcome assessors and data analysts being blinded, so unblinding will not occur. On the other hand, due to the nature of the intervention, the participants will know the allocation of the school.

## Data collection and management

### Plans for assessment and collection of outcomes {18a}

Data will be retrieved from:

1. The European Drug Addiction Prevention Trial Questionnaire (EU-Dap), a self-reported questionnaire, aims to screen substance use and assess risk and protective factors among adolescents. It has 45 multi-item questions and focuses especially on tobacco, alcohol, and marijuana use. The EU-Dap instrument was developed by the research team of the Unplugged program in Europe. It was used to evaluate the Unplugged trial project, which took place between 2003 and 2005 in seven countries in Europe. In the Chilean validation study, all subscales had acceptable internal reliability (omega > 0.65) [40]. The original form of the questionnaire can be found at http://www.eudap.net.
2. The Social Problem-Solving Inventory-Revised (SPSI-RS) consists of five scales that evaluate two dimensions of Problem Orientation (Positive Problem Orientation and Negative Problem Orientation) and three Troubleshooting styles (Rational Troubleshooting Style, Impulsive/Careless Style, and Avoidance Style). The abbreviated 25-item version [41] will be used in this study; in this shorter instrument, each scale has five items, with the full scale comprising 25 items. The inventory must be answered as a 5-choice Likert-like scale, in which the subjects indicate to what extent the proposed coping strategy applies to their characteristic way of dealing with the problems of daily life (0- it does not apply at all to me, 1- it applies slightly to me, 2- it applies moderately to me, 3- it applies a lot to me, and 4- it applies extremely to me). It has acceptable internal reliability (Cronbach alpha > 0.75) [41].
3. The Emotion Regulation Questionnaire for Children and Adolescents (ERQ-CA). Emotional regulation is defined as the ability to control and manage one’s feelings and emotions and appears to be an important factor in social adjustment. The questionnaire consists of 10 items. The answers on the questionnaire have a five-point Likert scale quantifying the agreement to the behavior described (1 = strongly disagree, 2 = disagree, 3 = half and half, 4 = agree, 5 = strongly agree). This instrument provides scores on two uncorrelated scales: Cognitive Reappraisal (6 items) that consists of redefining a potential emotion-eliciting situation in a way that its emotional impact is changed; and Expressive Suppression (4 items), encompassing a style that consists of the inhibition of ongoing emotion expressive behavior. It has acceptable internal reliability (Cronbach alpha > 0.75) [43].
4. The Strengths and Difficulties Questionnaire (SDQ). This is a questionnaire of 25 mental health items for a population of 4 to 17 years. It evaluates positive and negative psychological aspects, grouped into five sub-scales: (1) emotional symptoms, (2) behavioral problems, (3) attention and hyperactivity problems, (4) social relationship problems, and (5) pro-social behavior. The first four sub-scales refer to difficulties, while the last sub-scale refers to strengths. Several studies have shown that the instrument has good psychometric properties; our research team has already conducted a validation study in early adolescents in Chile [44]. This validation study confirmed the 5-dimensional structure of the instrument while also showing that its internal reliability ranges from 0.53 to 0.71 for the self-reporting version.
5. The Psychological Sense of School Membership Scale (PSSM). The sense of school membership refers to the student’s perceptions of the respect and acceptance that teachers and other students show them and their sense of belonging. It is a scale that our research team has already validated in Chile in early adolescents. This scale has 13 items (Chilean version) and is a single dimension, with high internal reliability (Cronbach’s alpha 0.92) [45].

### Plans to promote participant retention and complete follow-up {18b}

The schools, students, and their families will receive extensive information about the study setup and requirements during the recruitment. This information will include and stress the importance of completion of follow-up. From the start of the implementation of the assessments and the lessons of “Mi Mejor Plan,” students will be reminded of the value of their active participation during the whole project. Throughout the follow-up period, the researchers will check responses and, if necessary, contact schools and participants to complete their follow-up.

### Data management {19}

After the participants have completed the questionnaires, the data will be entered into a secure platform without identifying information (each participant will be assigned an encrypted ID number). The original copies of the instruments will be filed and stored, under lock and key, in a self-storage, along with the list linking the participants’ names and ID numbers. Only the Principal Investigator, research assistants in charge of data entry, and the statistician will have access to the database. All people with access to the dataset will need to sign a Confidential Agreement to assure its commitment to not revealing identifying information.

### Confidentiality {27}

Research data will be stored using a study identification code for each participant. The key to the identification code list will only be available to the research team mentioned above and will be documented and safeguarded according to research guidelines after completion of the study. No participant identification details will be reported in publications or any study report.

### Plans for collection, laboratory evaluation, and storage of biological specimens for genetic or molecular analysis in this trial/future use {33}

Not applicable. No biological specimens will be collected in this trial.

## Statistical methods

### Statistical methods for primary and secondary outcomes {20a}

General school features (size, number of teachers, etc.) will be used to compare participating schools with those who were invited but did not participate. Additionally, descriptive statistics will compare the two arms at baseline.

Primary and secondary analysis will be conducted on an intention-to-treat basis. Odds ratios (OR) and their corresponding confidence intervals (95%CI) will be calculated as the measure of association between experimental condition (intervention arm) and behavioral outcome, controlling for baseline outcome variable scores. Secondary analysis will be conducted considering adjustment for variables with marked imbalance at baseline. Additionally, secondary analyses will be made using the secondary outcomes and analyzed with the same approach. Considering the hierarchical structure of the data and the cluster effect, a multilevel modeling approach will be followed to analyze the data. Data will be analyzed with Stata 17.0.

### Interim analyses {21b}

Not applicable. There will not be interim analyses because the data will be analyzed at the end of the trial.

### Methods for additional analyses (e.g., subgroup analyses) {20b}

Subgroup analyses will be performed to explore differences between males and females. The statistical approach will follow the procedures described above.

### Methods in analysis to handle protocol non-adherence and any statistical methods to handle missing data {20c}

The primary outcome will be assessed using an intention-to-treat analysis. Missing data will be reduced to a minimum by using the appropriate measures: scourging students to fill out the whole questionnaire, a research assistant will revise the questionnaire when students end the evaluation and ask to complete the instruments if some questions are unanswered. Multiple imputations will be used to handle missing data in the primary and secondary analyses.

### Plans to give access to the full protocol, participant-level data and statistical code {31c}

The datasets produced during the current study will be available in an international database repository called UK Data Service.

## Oversight and monitoring

### Composition of the coordinating center and trial steering committee {5d}

This is a monocenter study designed, performed, and coordinated in Universidad de los Andes, Chile. Daily support for the trial is provided by the principal investigator, who supervises the trial. Additionally, a data manager organizes data collection and assures data quality. The study coordinator will help with the trial registration and coordinate study visits and reports. Study research assistants will help identify potential participating schools, collect informed consents, ensure follow-up according to protocol, and resolve doubts from school staff or participants.

The study team will meet weekly during the whole duration of the study. There is no trial steering committee or stakeholder and public involvement group. The Ethical Scientific Committee of the Universidad de los Andes will check the presence and completeness of the investigation.

### Composition of the data monitoring committee, its role, and reporting structure {21a}

A monitor from the Ethical Scientific Committee of the Universidad de los Andes will check the presence and completeness of the investigation once a year. This committee is independent of the sponsor and has no competing interests; further details about its charter can be asked via email: cec@uandes.cl.

### Adverse event reporting and harms {22}

The intervention or the data collection procedure does not infer harm among the participants. However, any situation that compromises participants’ physical and psychological integrity occurring during all different actions related to the project will be registered in a pre-design form. The Principal Investigator and Project Coordinator will manage this information, and, if necessary, they will contact school authorities, main caregivers, and local health providers. The whole research team will be trained in the Principles and the Code of Federal Regulations (CFR) for clinical research trials in the US [46] and will get the certification provided by the National Institute on Drug Abuse (NIDA). See https://gcp.nidatraining.org.

### Frequency and plans for auditing trial conduct {23}

A monitor from the Ethical Scientific Committee of the Universidad de los Andes will check once a year the presence and completeness of the investigation files, such as informed consents, inclusion and exclusion criteria, and data collection and storage.

### Plans for communicating important protocol amendments to relevant parties (e.g., trial participants, ethical committees) {25}

All substantial amendments will be notified to the ethics committee of the Universidad de los Andes. In case amendments concern or affect participants in any way, they will be informed about the changes. If needed, additional consent will be requested and registered. Also, online trial registries will be updated accordingly.

### Dissemination plans {31a}

The results of this research will be disclosed completely in international peer-reviewed journals. Both positive and negative results will be reported. An executive summary of the results will be given to school authorities.

## Discussion

The proposed study is the first to test the effectiveness of SCPP-YA and its substance use prevention module in Chile in a cluster randomized controlled trial (cRCT), and the first study evaluating the SCPP-YA program in Spanish-speaking Latin American countries.

Few studies have been conducted in Chile to examine the effectiveness of interventions to prevent substance use in early adolescence. Nevertheless, several initiatives have succeeded in developing socio-emotional skills in preadolescent school students [47, 48]. First, we expect that this study may provide more evidence about the effectiveness of substance use prevention programs aimed at adolescents; second, we hope that this Spanish-language version of “SCPP-YA” will become a free-of-charge resource for schools all over the country to implement.

Schools constitute especially fertile grounds for training large groups of children through preventive programs. Interventions such as Roger Weissberg’s SCPP-YA program, apart from bolstering children’s coping and adaptation skills, can also improve the social climate in the classroom, making it possible for students to learn more effectively and safely. Additionally, interventions of this type have also been shown to allow students to generate a stronger attachment and a deeper sense of belonging to their school, increasing engagement in healthy behaviors and reducing the likelihood of developing risk behaviors [25].

The SCPP-YA program has been implemented successfully in the USA. Thus, if the program effects are positive, wide implementation in Chile and other Latin American countries is possible in the near future. There are, however, some potential limitations. First, there might be an unknown impact of the COVID-19 pandemic on adolescents’ substance use, which may decrease substance use prevalence due to the isolation during lockdowns [49]. Second, there is a risk of low implementation quality in schools recruited into the cRCT. To avoid this threat, we will arrange face-to-face training sessions with the school personnel to motivate them to implement the SCPP-YA (Mi Mejor Plan) program as intended and ongoing support and coaching during the implementation process. Finally, there might be a risk of difficulty in recruiting enough schools into the cRCT. To minimize this risk, we will prepare the recruitment carefully and inform the schools in good time, using the excellent networks of the research team members.

## Trial status

Recruiting will start in January 2022. The current protocol is version 1 of 04-08-2021. Participants recruitment is estimated to be completed at the end of April 2022. Trial identifier NCT04236947 in Clinical Trials [ClinicalTrials.gov].

## Data Availability

The final trial dataset is planned to be available in an international data repository called UK Data Service.

## Abbreviations

SCPP-YA: Social Competence Promotion Program among Young Adolescents
cRCT: Cluster randomized clinical trial
EU-Dap: European Drug Addiction Prevention Trial Questionnaire
SENDA: National Service for the Prevention and Rehabilitation of Drugs and Alcohol
CONACE: National Council for Narcotics Control
PAHO: Pan American Health Organization
LSE: Life Skills Training Program
TAS: Treatment-As-Usual
IVE-SINAE: School Vulnerability Index – National System of Equality Allocation
SPSI-RS: Social Problem-Solving Inventory-Revised
ERQ-CA: Emotion Regulation Questionnaire for Children and Adolescents
SDQ: Strengths and Difficulties Questionnaire PSSM Psychological Sense of School Membership Scale
ICC: Intraclass correlation coefficient
CFR: Code of Federal Regulations
NIDA: National Institute on Drug Abuse
